# Predictive impact of fibrinogen-to-albumin ratio (FAR) for left ventricular dysfunction in acute coronary syndrome: a cross-sectional study

**DOI:** 10.1186/s40001-023-01029-2

**Published:** 2023-02-08

**Authors:** Xuan Wang, Yi Hu, Hao Luan, Chaodi Luo, Kamila·Kamili, Tingting Zheng, Gang Tian

**Affiliations:** Department of Cardiology, First Affiliated Hospital of Xi’an Jiaotong University, Xi’an, China

**Keywords:** Fibrinogen-to-albumin ratio, Left ventricular ejection fraction, Left ventricular systolic dysfunction, Inflammation, Acute coronary syndrome

## Abstract

**Background:**

The significantly prognostic value of fibrinogen-to-albumin ratio (FAR) has been proved in patients with coronary artery disease and different oncologic disorders. This study aimed to investigate the predictive value of FAR for left ventricular systolic dysfunction (LVSD) in acute coronary syndromes (ACS) patients.

**Methods:**

A total of 650 ACS patients after percutaneous coronary intervention (PCI) were eventually enrolled in the analysis. Participants were classified into three groups according to baseline FAR levels (T1: FAR < 73.00; T2: 73.00 ≤ FAR < 91.00; T3: FAR ≥ 91.00). The association between FAR and LVSD was assessed by binary logistic regression analysis. A nomogram to predict the risk of LVSD was constructed based on the output indices from multivariate regression analyses.

**Results:**

Patients with LVSD showed significantly higher FAR, monocyte-to-lymphocyte ratio (MLR), neutrophil-to-lymphocyte ratio (NLR), and platelet-to-lymphocyte ratio (PLR) than those without. FAR was an independent predictor of left ventricular dysfunction from the multivariate analyses (OR, 1.038; 95%CI, 1.020–1.057; *P* < 0.001). The area under receiver operating characteristic curve (AUC) of FAR predicting the occurrence of LVSD was 0.735. Meanwhile, FAR was the most powerful predictor than MLR, NLR, and PLR. Nomogram with the AUC reaching 0.906 showed a robust discrimination.

**Conclusions:**

Admission FAR is independently and significantly associated with LVSD in patients with ACS undergoing PCI.

**Supplementary Information:**

The online version contains supplementary material available at 10.1186/s40001-023-01029-2.

## Background

Acute coronary syndrome (ACS) remains the leading cause of morbidity and mortality worldwide despite prolonged and rigorous cardiovascular risk factor management [1]. Left ventricular systolic dysfunction (LVSD) is a common and serious complication of acute myocardial infarction (AMI), which can lead to greatly increased risks of sudden death and heart failure (HF) [2]. LVSD remains a major prognostic indicator for adverse cardiovascular events in patients with coronary artery disease (CAD) [3]. The presentation of left ventricular dysfunction shows a significant impact on the prognosis of ACS patients. Left ventricular ejection fraction (LVEF) is a conventional parameter to evaluate left ventricular systolic function in clinical practice and has been recognized as a significantly independent predictor of mortality in patients with ACS [4, 5]. In this context, the evaluation of clinical biomarkers associated with the occurrence of left ventricular dysfunction for further optimal management is considered to improve risk stratification in ACS patients.

Fibrinogen-to-albumin ratio (FAR) is measured by dividing serum fibrinogen by serum albumin. Both fibrinogen and albumin are reliable indicators of chronic systemic inflammation. Inflammation plays a crucial part in the initiation and progression of the atherosclerotic plaque rupture, thrombus formation and endothelial dysfunction [6]. Several studies have demonstrated that inflammatory biomarkers, including neutrophil-to-lymphocyte ratio (NLR) [7], platelet-to-lymphocyte ratio (PLR) [8], monocyte-to-lymphocyte ratio (MLR) [9], fibrinogen [10] and albumin [11], correlate with the prognosis of ACS. However, the predictive role of FAR in occurrence of left ventricular dysfunction in ACS patients is still indistinct. This study aims to explore the significance of FAR on the occurrence of LVSD, so as to provide insights for the role of inflammation in the deterioration of left ventricular function in patients with ACS. Moreover, We aim to compare the predictive value of FAR, NLR, PLR, and MLR for LVSD to provide instructions for clinical treatment of ACS patients.

## Methods

### Participants

**Fig. 1:**
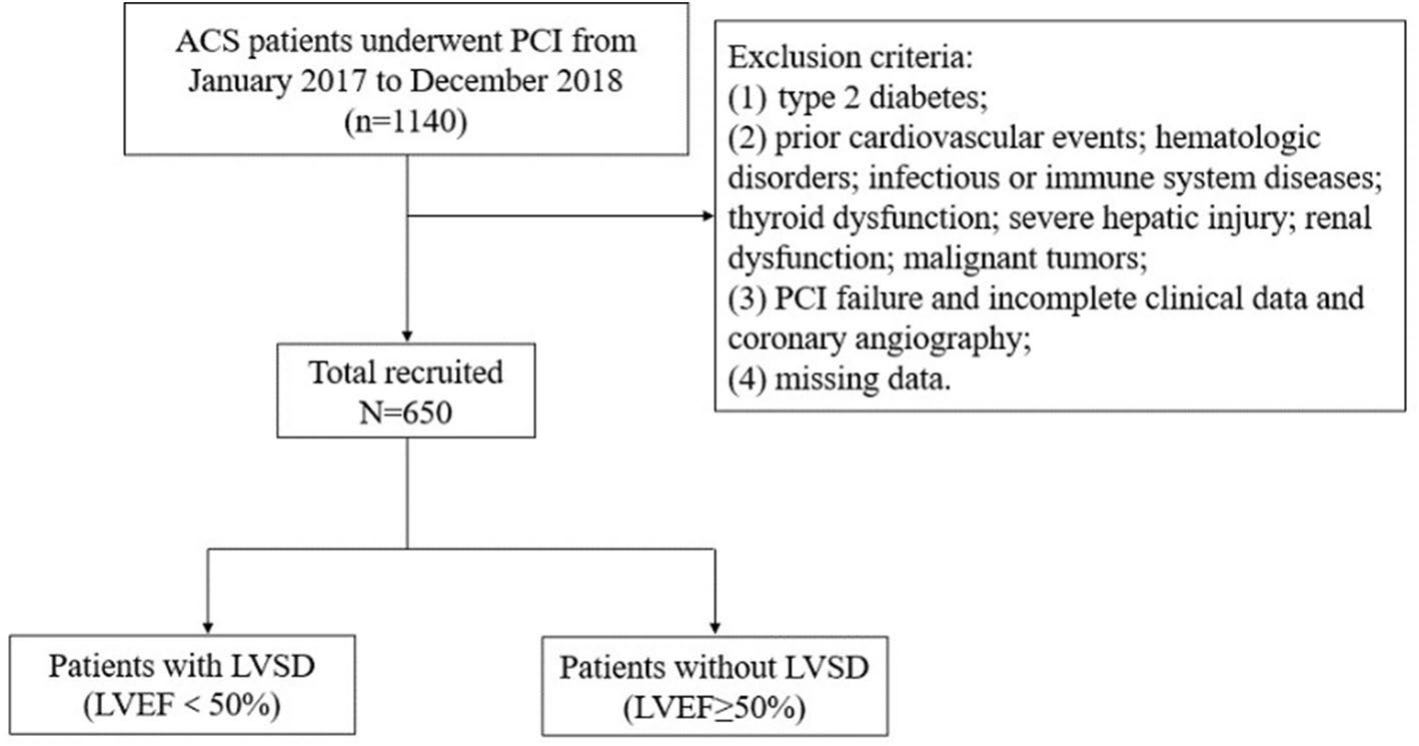
Flowchart of the study population enrollment. *ACS* acute coronary syndromes, *LVEF* left ventricular ejection fraction, *LVSD* left ventricular systolic dysfunction, *PCI* percutaneous coronary intervention

Patients who were diagnosed with ACS and underwent percutaneous coronary intervention (PCI) were consecutively enrolled from January 2017 and December 2018 at the First Affiliated Hospital of Medical College of Xi’an Jiaotong University in this single-center, retrospective, observational cohort study. The inclusion criteria were as follows: (1) age ≥ 18 years; (2) diagnosis of ACS, including unstable angina (UA), non-ST-segment elevation myocardial infarction (NSTEMI) and ST-segment elevation myocardial infarction (STEMI); (3) treated with elective PCI. The exclusion criteria included patients with prior cardiovascular events; type 2 diabetes; severe hepatic injury; hematologic disorders; acute infection; immune system diseases; thyroid dysfunction; renal insufficiency or chronic dialysis; malignant tumors; pregnancy; PCI failure; incomplete clinical and angiographic data. Ultimately, a cohort of 650 patients based on strict inclusion and exclusion criteria were enrolled (Fig. 1). This retrospective study obtained the ethical approval from the Ethical Committee of the First Affiliated Hospital of Xi’an Jiaotong University and was performed in accordance with the principles of the Declaration of Helsinki.

### Clinical data collection

Baseline data of demographic characteristics, including age, gender, weight, height, smoking, drinking, family history, and medication use were extracted from the standard medical records. BMI was calculated as weight in kilograms divided by squared height in meters (kg/m^2^). Heart rate and blood pressure measurements on admission was recorded. Patients with repeated systolic blood pressure ≥ 140 mmHg and/or diastolic blood pressure ≥ 90 mmHg, or receiving anti-hypertensive agents were considered criteria for hypertension [12]. Smoking was defined as an individual smoked a cigarette in the past 30 days or > 100 cigarettes in lifetime. Family history of CAD was defined as the occurrence of CAD in a first-degree relative. The routine hematology and biochemical parameters for baseline laboratory tests were drawn from the antecubital vein on admission and on the second day of hospitalization after an 8-h fast overnight. The Gensini score was calculated according to the results of coronary angiography. PCI was conducted in accordance with existing practice guidelines in China [13].

### Definition of inflammatory markers

FAR is the ratio between serum fibrinogen and serum albumin. NLR is calculated by dividing neutrophil count by lymphocyte count. PLR is defined as the ratio of the platelet value and lymphocyte value. The ratio of the monocyte value and lymphocyte value means MLR.

### Endpoint

LVEF was assessed using an ultrasonic cardiogram by two-dimensional Simpson’s method to determine the left ventricular systolic function. Patients were categorized into two groups based on their LVEF at 24 h after admission. Preserved systolic function was defined as LVEF ≥ 50% (*n* = 389) and LVSD was defined as LVEF < 50% (*n* = 261).

### Statistical analysis

Continuous variables were, respectively, expressed as the mean ± standard deviation or median (interquartile ranges) according to whether normal distribution or not, while categorical variables were presented as percentages. The Kolmogorov–Smirnov test was used to analyze the normality of distribution. Student’s *t* test was used for comparison of continuous variables with normal distribution, and asymmetrically distributed variables were compared by Mann–Whitney *U* test, while percentages were analyzed by the Chi-squared test. The correlations between FAR and traditional cardiovascular risk factors were evaluated by adopting the Spearman’s rank correlation test or Pearson correlation test when variables appropriate. The receiver operating characteristic (ROC) curve was drawn to evaluate the diagnostic efficiency of inflammatory indicators for LVSD by determining the value of the area under the ROC curve (AUC) and the optimal cut-off values was counted according to the maximum Youden index. The predictive value of the FAR for LVEF was assessed by univariate and multivariate logistic regression model. Predictors of the endpoint determined by univariate analysis, potential confounders, and clinical importance were all included in multivariate analysis. Further subgroup analyses according to gender, age (< 65 and ≥ 65 years), hypertension, BMI (< 25 and ≥ 25 kg/m^2^), and diagnosis (NSTE-ACS and STEMI) were employed to examine the consistence of the prediction of FAR for LVSD. The performance of the nomogram was assessed by calibration and decision curve analyses (DCA). Statistical analyses were conducted using SPSS software version 23.0 and R 3.1.2. All the statistics are two-tailed and *P* < 0.05 was considered to be statistically significant.

## Results

### Patient characteristics

The baseline characteristics of enrolled patients stratified by the occurrence of LVSD (left ventricular systolic dysfunction) at admission are illustrated in Table 1. A total of 650 patients (age: 61.63 ± 10.57 years; 77.2% men) were finally enrolled in present study. Compared with those without LVSD, patients with systolic dysfunction had lower systolic blood pressure, higher heart rate and higher prevalence of smoking. Patients with LVSD presented higher level of NT-proBNP, white blood cells, NLR, MLR, PLR, hs-CRP, ALT, AST, creatinine, HCY, FAR, INR, APTT, FIB, d-dimer, FDP as well as higher prevalence of STEMI diagnosis but lower levels of albumin and apolipoprotein A. As for the angiographic findings, patients with LVSD were more likely to have three-vessel disease and significantly higher Gensini score. While there was no significant difference considering body mass index, age, drinking habits, hypercholesterolemia and FBG.

**Table 1:** Baseline clinical and procedure characteristics of patients according to ejection fraction

Baseline clinical characteristics	Total population (n = 650)	LVEF < 50% (*n* = 261)	LVEF ≥ 50% (*n* = 389)	*P* value
Age, years	61.63 ± 10.57	62.08 ± 10.66	61.32 ± 10.51	0.418
Sex, male, *n* (%)	502 (77.2)	217 (83.1)	285 (73.3)	0.003
BMI, kg/m^2^	25.28 ± 3.19	25.19 ± 3.35	25.35 ± 3.04	0.427
Heart rate, bpm	74 (66–83)	78 (68–89)	72 (66–80)	< 0.001
SBP, mmHg	129.93 ± 19.75	125.21 ± 20.77	133.10 ± 18.39	< 0.001
DBP, mmHg	79.79 ± 13.18	79.96 ± 14.58	79.68 ± 12.17	0.940
Smoking, *n* (%)	350 (53.8)	166 (63.6)	184 (47.3)	< 0.001
Drinking, *n* (%)	126 (19.4)	47 (18.0)	79 (20.3)	0.467
Hypertension, *n* (%)	359 (55.2)	121 (46.4)	238 (61.2)	< 0.001
Family history of CAD, *n* (%)	71 (10.9)	22 (8.4)	49 (12.6)	0.095
NT-proBNP, pg/mL	281.2 (92.2–1069.0)	1075.5 (351.0–2840.0)	132.5 (56.14–367.05)	< 0.001
Cardiac troponin T, ng/mL	0.305 (0.009–0.492)	0.418 (0.044–1.575)	0.110 (0.007–0.049)	< 0.001
Hemoglobin, g/L	141.48 ± 16.46	141.19 ± 17.32	141.68 ± 15.88	0.778
Platelet, 10^9^/L	205.38 ± 63.37	206.43 ± 67.71	204.68 ± 60.36	0.851
White blood cells, 10^9^/L	7.15 (5.61–9.47)	8.40 (6.38–10.96)	6.57 (5.32–8.35)	< 0.001
Neutrophils, 10^9^/L	4.97 (3.66–7.09)	6.55 (4.34–8.86)	4.43 (3.40–5.87)	< 0.001
Lymphocyte, 10^9^/L	1.43 (1.07–1.86)	1.31 (0.98–1.79)	1.52 (1.15–1.91)	< 0.001
Monocytes, 10^9^/L	0.35 (0.28–0.47)	0.41 (0.30–0.56)	0.33 (0.26–0.43)	< 0.001
NLR	3.29 (2.31–5.71)	4.79 (2.84–7.50)	2.88 (2.06–4.05)	< 0.001
MLR	0.24 (0.18–0.34)	0.28 (0.22–0.42)	0.21 (0.16–0.29)	< 0.001
PLR	136.47 (104.06–185.04)	149.31 (110.22–203.71)	129.82 (97.81–169.92)	< 0.001
hs-CRP, mg/L	1.90 (0.77–5.40)	3.59 (1.54–10.00)	1.145 (0.54–3.07)	< 0.001
ALT, U/L	27 (18–40)	30 (21–47)	23 (16–35)	< 0.001
AST, U/L	26 (20–56)	47 (26–116)	22 (18–31)	< 0.001
Albumin, g/L	40.54 ± 4.65	38.86 ± 4.88	41.67 ± 4.13	< 0.001
BUN, mmol/L	5.66 ± 1.78	5.77 ± 2.09	5.58 ± 1.54	0.992
Scr, µmol/L	67.78 ± 19.59	70.35 ± 23.19	66.05 ± 16.54	0.047
Cystatin C, mg/L	1.032 ± 0.326	1.079 ± 0.336	1.001 ± 0.316	0.005
FPG, mg/dL	4.74 (4.20–5.42)	4.70 (4.27–5.44)	4.77 (4.20–5.41)	0.966
RBG, mg/dL	6.23 (5.31–7.68)	6.23 (5.33–7.65)	6.23 (5.29–7.71)	0.776
eGFR, mL/(min*1.73 m^2^)	96.87 (88.69–104.62)	95.77 (85.58–104.05)	97.62 (90.35–105.52)	0.058
K^+^, mmol/L	3.93 ± 0.39	3.92 ± 0.39	3.93 ± 0.40	0.629
Na^+^, mmol/L	141.04 ± 3.39	140.24 ± 3.92	141.57 ± 2.87	< 0.001
Ca^2+^, mmol/L	2.30 ± 0.14	2.25 ± 0.14	2.33 ± 0.13	< 0.001
Uric acid, µmol/L	340.25 ± 86.98	339.28 ± 91.75	340.91 ± 83.73	0.763
Homocysteine, µmol/L	17.7 (13.9–23.5)	20.7 (15.8–33.4)	15.9 (13.3–20.4)	< 0.001
PT, s	13.4 (13.0–13.8)	13.7 (13.2–14.1)	13.3 (12.9–13.7)	< 0.001
PTA, %	90.94 ± 13.64	87.83 ± 13.62	93.03 ± 13.27	< 0.001
INR	1.04 (1.00–1.08)	1.06 (1.02–1.11)	1.03 (0.99–1.07)	< 0.001
APTT, s	36.4 (33.9–39.4)	37.7 (34.7–41.4)	35.9 (33.4–38.3)	< 0.001
TT, s	16.6 (15.8–17.4)	16.6 (15.7–17.5)	16.5 (15.9–17.3)	0.727
FIB, g/L	3.33 (2.84–3.79)	3.58 (3.12–4.32)	3.15 (2.69–3.53)	< 0.001
d-dimer, mg/L	0.44 (0.30–0.70)	0.56 (0.40–0.90)	0.40 (0.30–0.56)	< 0.001
FDP, mg/L	1.20 (0.90–1.70)	1.40 (0.96–2.30)	1.20 (0.90–1.50)	< 0.001
Triglycerides, mmol/L	1.29 (0.97–1.82)	1.17 (0.83–1.66)	1.38 (1.04–1.97)	< 0.001
TC, mmol/L	3.74 (3.14–4.42)	3.77 (3.12–4.46)	3.73 (3.15–4.38)	0.638
LDL, mmol/L	2.21 (1.68–2.79)	2.28 (1.71–2.79)	2.15 (1.65–2.79)	0.242
HDL, mmol/L	0.91 (0.78–1.07)	0.92 (0.78–1.06)	0.91 (0.77–1.08)	0.884
apoA, g/L	1.082 (0.967–1.212)	1.067 (0.918–1.194)	1.100 (0.995–1.232)	0.001
apoB, g/L	0.763 (0.627–0.924)	0.800 (0.627–0.934)	0.751 (0.622–0.909)	0.202
apoE, g/L	33.1 (26.5–40.8)	32.5 (26.7–41.1)	33.4 (26.3–40.7)	0.877
Lp (a), mg/L	184 (95–338)	239 (118–371)	153 (86–309)	< 0.001
LVEF, %	60 (45–67)	43 (39–47)	66 (62–70)	< 0.001
FAR	81.43 (67.66–97.62)	91.37 (79.38–116.50)	75.14 (63.76–87.13)	< 0.001
Gensini score	62 (40–90)	80 (50–100)	52 (34–80)	< 0.001
Initial diagnosis, *n* (%)				0.138
UA	352 (54.2)	59 (22.6)	293 (75.3)	< 0.001
NSTEMI	82 (12.6)	41 (15.7)	41 (10.5)	0.052
STEMI	216 (33.2)	161 (61.7)	55 (14.1)	< 0.001
Killip class				
I	285 (43.8)	165 (63.2)	120 (30.8)	< 0.001
II	303 (46.6)	57 (21.8)	246 (63.2)	< 0.001
≥ III	62 (9.5)	39 (14.9)	23 (5.9)	< 0.001
Diseased vessels number, *n* (%)				
One-vessel disease	146 (12.9)	142 (13.8)	4 (3.9)	0.005
Two-vessel disease	316 (28.0)	294 (28.6)	22 (21.6)	0.131
Three-vessel disease	663 (58.7)	587 (57.1)	76 (74.5)	< 0.001
Diseased vessels type, *n* (%)				
LM	85 (7.5)	76 (7.4)	9 (8.8)	0.601
LAD	1055 (93.4)	958 (93.2)	97 (95.1)	0.460
LCX	845 (74.8)	770 (74.9)	75 (73.5)	0.761
RCA	860 (76.1)	779 (75.8)	81 (79.4)	0.412
Target vessel territory, *n* (%)				
LAD	742 (65.7)	677 (65.9)	65 (63.7)	0.666
LCX	356 (31.5)	325 (31.6)	31 (30.4)	0.800
RCA	482 (42.7)	444 (43.2)	38 (37.3)	0.248
Number of stents, *n* (%)				
1	404 (35.8)	365 (35.5)	39 (38.2)	0.583
2	341 (30.2)	320 (31.1)	21 (20.6)	0.027
≥ 3	385 (34.1)	343 (33.4)	42 (41.2)	0.112
Average length of stents, mm	26.79 ± 5.86	26.73 ± 5.84	27.39 ± 6.08	0.321
Average width of stents, mm	2.98 ± 0.43	2.98 ± 0.42	2.98 ± 0.43	0.652
Plaque property, *n* (%)				
Calcification lesions	142 (21.8)	45 (17.2)	97 (24.9)	0.020
Diffuse lesions	171 (26.3)	56 (21.5)	115 (29.6)	0.021
Thrombus	30 (4.6)	18 (6.9)	12 (3.1)	0.023
Chronic total occlusions	93 (14.3)	51 (19.5)	42 (10.8)	0.002

Data are presented as the IQR, mean ± SD or *n* (%)

*BMI* body mass index, *SBP* systolic blood pressure, *DBP* diastolic blood pressure, *CAD* coronary artery disease, *hs-CRP* high-sensitivity C-reactive protein, *NT-proBNP* N-terminal pro-B type natriuretic peptide, *ALT* alanine transaminase, *AST* aspartate aminotransferase, *BUN* blood urea nitrogen, *SCr* serum creatinine concentration, *FPG* fasting plasma glucose, *RBG* random blood sugar, *HbA1c* glycosylated hemoglobin A1c, eGFR estimated glomerular filtration rate,* K*^+^ serum potassium, *Na*^+^ serum sodium, *Ca*^*2*+^ serum calcium, *PT* prothrombin time, *PTA* prothrombin time activity, *INR* international normalized ratio, *APTT* activated partial thromboplastin time, *TT* thrombin time, *FIB* fibrinogen, *FDP* fibrinogen degradation products, *TC* total cholesterol, *LDL-C* low-density lipoprotein cholesterol, *HDL-C* high-density lipoprotein cholesterol, *apoA* apolipoprotein A, *apoB* apolipoprotein B, *apoE* apolipoprotein E, *Lp(a)* Lipoprotein(a), *LVEF* left ventricular ejection fraction, *UA* unstable angina, *NSTEMI* non-ST-segment elevation myocardial infarction, *STEMI* ST-segment elevation myocardial infarction, *LM* left main artery, *LAD* left anterior descending artery, *LCX* left circumflex artery, *RCA* right coronary artery

Baseline clinical and procedure characteristics of patients categorized by the FAR tertiles are presented in Table 2. Patients with high FAR seemed to be older and higher heart rate. Laboratory indexes including NT-proBNP, cardiac troponin T, white blood cells, NLR, MLR, PLR, hs-CRP, cystatin C, FIB, FDP and FAR increased, whereas SBP, DBP, hemoglobin, triglycerides and LVEF decreased in proportion to the FAR tertiles. The rate of smoking, drinking, hypertension, family history of CAD and BMI level were not different among the different FAR groups. In the top FAR tertile, most patients were diagnosed as STEMI and showed significantly higher Gensini score.

**Table 2:** Baseline clinical and procedure characteristics of patients stratified by the FAR tertiles

Baseline clinical characteristics	T1 (*n* = 216)	T2 (*n* = 219)	T3 (*n* = 215)	*P* value
Age, years	59.54 ± 10.88	62.32 ± 9.59	63.01 ± 10.93	0.001
Sex, male, *n* (%)	174 (80.6)	169 (77.2)	159 (74.0)	0.263
BMI, kg/m^2^	25.46 ± 3.08	25.20 ± 3.35	25.19 ± 3.13	0.558
Heart rate, bpm	72 (64–81)	74 (66–82)	76 (68–86)	0.003
SBP, mmHg	132.2 ± 17.9	131.0 ± 20.7	126.5 ± 20.0	0.002
DBP, mmHg	81.3 ± 12.6	80.3 ± 13.2	77.7 ± 13.4	0.012
Smoking, *n* (%)	110 (50.9)	118 (53.9)	122(56.7)	0.480
Drinking, *n* (%)	39 (18.1)	50 (22.8)	37(17.2)	0.278
Hypertension, *n* (%)	124 (57.4)	117 (53.4)	118(54.9)	0.700
Family history of CAD, *n* (%)	27 (12.5)	23 (10.5)	21(9.8)	0.642
NT-proBNP, pg/mL	109.00 (53.53–286.40)	263.25 (95.99–756.55)	1020.00(297.85–3054.00)	< 0.001
Cardiac troponin T, ng/mL	0.012 (0.007–0.056)	0.040 (0.009–0.402)	0.198(0.015–1.485)	< 0.001
Hemoglobin, g/L	144.87 ± 14.80	143.41 ± 15.66	136.12 ± 17.54	< 0.001
Platelet, 10^9^/L	199.80 ± 61.37	198.35 ± 49.99	218.16 ± 74.84	0.016
White blood cells, 10^9^/L	6.89 (5.36–9.08)	6.81 (5.50–9.40)	7.59(5.98–9.94)	0.012
Neutrophils, 10^9^/L	4.82 (3.46–6.60)	4.86 (3.56–7.00)	5.47(4.09–7.57)	0.008
Lymphocyte, 10^9^/L	1.44 (1.07–1.86)	1.43 (1.11–1.85)	1.45(1.04–1.87)	0.853
Monocytes, 10^9^/L	0.32 (0.25–0.41)	0.34 (0.28–0.44)	0.42(0.31–0.57)	< 0.001
NLR	3.10 (2.17–4.60)	3.28 (2.25–5.93)	3.61(2.51–6.23)	0.029
MLR	0.22 (0.16–0.28)	0.24 (0.18–0.32)	0.29(0.19–0.43)	< 0.001
PLR	131.37 (101.45–171.25)	134.61 (99.48–179.41)	140.35(106.66–203.29)	0.040
hs-CRP, mg/L	0.78 (0.39–1.85)	1.77 (0.90–3.93)	5.77(2.86–10.00)	< 0.001
ALT, U/L	25 (18–39)	27 (18–38)	26(18–44)	0.001
AST, U/L	23 (19–33)	26 (20–57)	31(20–73)	0.777
Albumin, g/L	43.44 ± 3.94	40.85 ± 3.89	37.31 ± 3.94	< 0.001
BUN, mmol/L	5.69 ± 1.52	5.52 ± 1.59	5.77 ± 2.18	0.390
Scr, µmol/L	67.19 ± 16.91	66.10 ± 17.21	70.08 ± 23.78	0.290
Cystatin C, mg/L	1.006 ± 0.318	1.012 ± 0.331	1.079 ± 0.326	0.033
FPG, mg/dL	4.64 (4.16–5.34)	4.85 (4.27–5.52)	4.74(4.18–5.37)	0.176
RBG, mg/dL	6.30 (5.31–7.84)	6.35 (5.45–7.73)	6.11(5.19–7.37)	0.131
eGFR, mL/(min*1.73 m^2^)	98.18 (90.43–106.43)	98.31 (90.76–104.44)	94.27(84.78–102.40)	0.001
K^+^, mmol/L	3.92 ± 0.37	3.92 ± 0.39	3.94 ± 0.42	0.884
Na^+^, mmol/L	141.33 ± 2.89	141.16 ± 3.01	140.62 ± 4.13	0.281
Ca^2+^, mmol/L	2.34 ± 0.12	2.31 ± 0.15	2.25 ± 0.14	< 0.001
Uric acid, µmol/L	347.16 ± 88.77	342.33 ± 87.12	331.18 ± 84.60	0.148
Homocysteine, µmol/L	16.4 (13.7–22.8)	17.9 (13.6–23.7)	18.0 (14.6–24.5)	0.224
PT, s	13.3 (12.9–13.7)	13.4 (13.0–13.8)	13.6 (13.2–14.1)	< 0.001
PTA, %	93.71 ± 12.73	92.07 ± 13.69	87.03 ± 13.63	< 0.001
INR	1.03 (0.99–1.07)	1.03 (0.99–1.07)	1.06 (1.02–1.11)	< 0.001
APTT, s	35.95 (33.1–38.6)	36.2 (33.9–39.4)	37.0 (34.4–40.7)	0.009
TT, s	16.8 (16.1–17.5)	16.6 (15.9–17.4)	16.1 (15.4–17.1)	< 0.001
FIB, g/L	2.66 (2.44–2.92)	3.33 (3.09–3.51)	4.10 (3.69–4.77)	< 0.001
d-dimer, mg/L	0.40 (0.30–0.50)	0.41 (0.30–0.60)	0.60 (0.40–1.10)	< 0.001
FDP, mg/L	1.00 (0.70–1.30)	1.20 (0.91–1.50)	1.60 (1.20–2.70)	< 0.001
Triglycerides, mmol/L	1.37 (1.09–1.92)	1.30 (0.99–1.88)	1.17 (0.85–1.63)	< 0.001
TC, mmol/L	3.80 (3.19–4.52)	3.73 (3.15–4.46)	3.59 (3.07–4.29)	0.223
LDL, mmol/L	2.28 (1.70–2.87)	2.22 (1.68–2.79)	2.05 (1.65–2.72)	0.176
HDL, mmol/L	0.93 (0.79–1.09)	0.91 (0.77–1.07)	0.90 (0.76–1.02)	0.138
apoA, g/L	1.136 (1.025–1.240)	1.083 (0.984–1.228)	1.035 (0.903–1.162)	< 0.001
apoB, g/L	0.770 (0.630–0.922)	0.751 (0.629–0.928)	0.757 (0.617–0.910)	0.907
apoE, g/L	33.9 (26.0–41.2)	32.5 (26.6–42.0)	32.5 (26.7–39.4)	0.703
Lp (a), mg/L	148 (88–301)	171 (84–333)	236 (121–378)	0.001
LVEF, %	64 (55–69)	62 (46–69)	47 (42–63)	< 0.001
FAR	62.80 (57.57–67.66)	81.46 (77.29–85.28)	107.14 (97.65–127.86)	< 0.001
Gensini score	52 (34–84)	62 (40–88)	72 (48–98)	0.001
Initial diagnosis, *n* (%)				
UA	147 (68.1)	122 (55.7)	83 (38.6)	< 0.001
NSTEMI	16 (7.4)	32 (14.6)	34 (15.8)	0.017
STEMI	53 (24.5)	65 (29.7)	98 (45.6)	< 0.001
Killip class, *n* (%)				
I	78 (36.1)	101 (46.1)	106 (49.3)	0.016
II	123 (56.9)	102 (46.6)	78 (36.3)	< 0.001
≥ III	15 (6.9)	16 (7.3)	31 (14.4)	0.012
Diseased vessels number, *n* (%)				
One-vessel disease	44 (20.4)	51 (23.3)	44 (20.5)	0.700
Two-vessel disease	69 (31.9)	49 (22.4)	49 (22.8)	0.036
Three-vessel disease	103 (47.7)	119 (54.3)	122 (56.7)	0.148
Diseased vessels type, *n* (%)				
LM	16 (7.4)	21(9.6)	21 (9.8)	0.632
LAD	202 (93.5)	199 (90.9)	208 (96.7)	0.042
LCX	142 (65.7)	144 (65.8)	145 (67.4)	0.912
RCA	147 (68.1)	161 (73.5)	152 (70.7)	0.457
Target vessel territory, *n* (%)				
LAD	130 (60.2)	147 (67.1)	141 (65.6)	0.285
LCX	61 (28.2)	76 (34.7)	66 (30.7)	0.340
RCA	113 (52.3)	103 (47.0)	98 (45.6)	0.338
Number of stents, *n* (%)				
1	90 (41.7)	88 (40.2)	75 (34.9)	0.316
2	66 (30.6)	64 (29.2)	76 (35.3)	0.355
≥ 3	60 (27.8)	67 (30.6)	64 (29.8)	0.803
Average length of stents, mm	27.61 ± 6.29	28.60 ± 5.83	27.47 ± 6.17	0.053
Average width of stents, mm	3.04 ± 0.45	2.99 ± 0.39	2.91 ± 0.44	0.016
Plaque property, *n* (%)				
Calcification lesions	47 (21.8)	52 (23.7)	43 (20.0)	0.640
Diffuse lesions	56 (25.9)	60 (27.4)	55 (25.6)	0.901
Thrombus	8 (3.7)	13 (5.9)	9 (4.2)	0.505
Chronic total occlusions	25 (11.6)	31 (14.2)	37 (17.2)	0.247

Data are presented as the IQR, mean ± SD or *n* (%)

*BMI* body mass index, *SBP* systolic blood pressure, *DBP* diastolic blood pressure, *CAD* coronary artery disease, *hs-CRP* high-sensitivity C-reactive protein, *NT-proBNP* N-terminal pro-B type natriuretic peptide, *FAR* fibrinogen-to-albumin ratio, *MLR* monocyte-to-lymphocyte ratio, *NLR* neutrophil-to-lymphocyte ratio, *PLR* platelet-to-lymphocyte ratio, *ALT* alanine transaminase, *AST* aspartate aminotransferase, *BUN* blood urea nitrogen, *SCr* serum creatinine concentration, *FPG* fasting plasma glucose, *RBG* random blood sugar, *HbA1c* glycosylated hemoglobin A1c, eGFR estimated glomerular filtration rate,* K*^+^ serum potassium, *Na*^+^ serum sodium, *Ca*^*2*+^ serum calcium, *PT* prothrombin time, *PTA* prothrombin time activity, *INR* international normalized ratio, *APTT* activated partial thromboplastin time, *TT* thrombin time, *FIB* fibrinogen, *FDP* fibrinogen degradation products, *TC* total cholesterol, *LDL-C* low-density lipoprotein cholesterol, *HDL-C* high-density lipoprotein cholesterol, *apoA* apolipoprotein A, *apoB* apolipoprotein B, *apoE* apolipoprotein E, *Lp(a)* Lipoprotein(a), *LVEF* left ventricular ejection fraction, *UA* unstable angina, *NSTEMI* non-ST-segment elevation myocardial infarction, *STEMI* ST-segment elevation myocardial infarction, *LM* left main artery, *LAD* left anterior descending artery, *LCX* left circumflex artery, *RCA* right coronary artery

### Correlation between FAR with LVEF and other cardiovascular risk factors

**Fig. 2:**
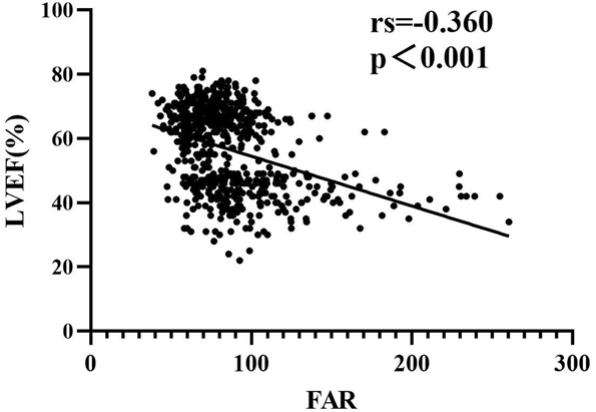
Scatter dot presentation comparison of FAR and LVEF. *LVEF* left ventricular ejection fraction, *FAR* fibrinogen-to-albumin ratio

Spearman correlation analysis revealed significantly negative associations between LVEF and FAR (*r* = − 0.360, *P* < 0.001) (Fig. 2). FAR was positively correlated with age, HR, NT-proBNP, cardiac troponin T, platelet, white blood cells, neutrophils, monocytes, NLR, MLR, PLR, hs-CRP, AST, cystatin C, PT, INR, APTT, D-dimer, FDP and Lp (a), while negatively correlated with SBP, DBP, eGFR, serum Na^+^, serum Ca^2+^, PTA, TT, triglycerides, HDL, apoA (Table 3).

**Table 3:** Correlations between FAR and traditional cardiovascular risk factors

	Correlation coefficient	*P* value
Age	0.152	< 0.001
Heart rate	0.152	< 0.001
SBP	− 0.143	< 0.001
DBP	− 0.120	0.002
NT-proBNP	0.514	< 0.001
Cardiac troponin T	0.382	< 0.001
Platelet	0.121	0.002
White blood cells	0.151	< 0.001
Neutrophils	0.160	< 0.001
Monocytes	0.274	< 0.001
NLR	0.138	< 0.001
MLR	0.259	< 0.001
PLR	0.113	0.004
hs-CRP	0.590	< 0.001
AST	0.184	< 0.001
Albumin	− 0.584	< 0.001
Cystatin C	0.105	0.008
eGFR	− 0.158	< 0.001
Na^+^	− 0.100	0.011
Ca^2+^	− 0.284	< 0.001
PT	0.222	< 0.001
PTA	− 0.205	< 0.001
INR	0.219	< 0.001
APTT	0.125	0.001
TT	− 0.217	< 0.001
FIB	0.902	< 0.001
D-dimer	0.416	< 0.001
FDP	0.458	< 0.001
Triglycerides	− 0.160	< 0.001
HDL	− 0.089	0.025
apoA	− 0.232	< 0.001
Lp (a)	0.181	< 0.001

*SBP* systolic blood pressure, *DBP* diastolic blood pressure, *hs-CRP* high-sensitivity C-reactive protein, *NT-proBNP* N-terminal pro-B type natriuretic peptide, *FAR* fibrinogen-to-albumin ratio, *MLR* monocyte-to-lymphocyte ratio, *NLR* neutrophil-to-lymphocyte ratio, *PLR* platelet-to-lymphocyte ratio, *AST* aspartate aminotransferase, *eGFR* estimated glomerular filtration rate, *Na*^+^ serum sodium, *Ca*^*2*+^ serum calcium, *PT* prothrombin time, *PTA* prothrombin time activity, *INR* international normalized ratio, *APTT* activated partial thromboplastin time, *TT* thrombin time, *FIB* fibrinogen, *FDP* fibrinogen degradation products, *apoA* apolipoprotein A, *Lp(a)* Lipoprotein(a)

**Table 4:** Predictive value of FAR for LVEF in different logistic regression analysis

	FAR as a continuous variable^a^		
	OR	95% CI	*P* value
Crude model	1.037	1.029–1.046	< 0.001
Model1	1.019	1.007–1.030	0.001
Model2	1.026	1.011–1.042	0.001
Model3	1.026	1.008–1.045	0.005
Model4	1.030	1.011–1.049	0.002

	FAR as a categorical variable^b^				
	T1	T2		T3	
		OR (95% CI)	*P* value	OR (95% CI)	*P* value
Crude model	Reference	2.628 (1.705–4.052)	< 0.001	6.854 (4.434–10.594)	< 0.001
Model1	Reference	2.090 (1.223–3.571)	0.007	2.140 (1.166–3.927)	0.014
Model2	Reference	2.431 (1.175–5.029)	0.017	3.699 (1.649–8.298)	0.002
Model3	Reference	2.530 (1.094–5.854)	0.030	3.738 (1.512–9.242)	0.004
Model4	Reference	2.105 (0.869–5.094)	0.099	3.395 (1.303–8.848)	0.012

Model 1: adjusted for age, sex (female), BMI, HR, SBP, DBP, smoking, hypertension, NT-proBNP, cardiac troponin T

Model 2: adjusted for variables included in Model 1 and white blood cells, NLR, MLR, PLR, hs-CRP, ALT, AST

Model 3: adjusted for variables included in Model 2 and cystatin C, Na^+^, Ca^2+^, homocysteine, PT, PTA, INR, APTT, d-dimer, FDP, triglycerides, apoA, Lp(a)

Model 4: adjusted for variables included in Model 3 and Gensini score, initial diagnosis (STEMI), Killip class (≥ III)

*OR* odds ratio, *CI* confidence interval

^a^The OR was examined by per 1-unit increase of FAR

^b^The OR was examined regarding T1 (the lowest) as reference

### The predictive implication of FAR

Univariate and multivariate logistic regression analyses and predictors of LVSD in ACS patients are presented in Additional file 1: Table S1. Univariate analyses showed that FAR, gender, HR, SBP, smoking history, hypertension, NT-proBNP, white blood cells, NLR, MLR, PLR, hs-CRP, ALT, AST, albumin, creatinine, cystatin C, eGFR, serum Na^+^, serum Ca^2+^, HCY, PT, PTA, INR, APTT, FIB, d-dimer, FDP, triglycerides, Lp(a), Gensini score, initial diagnosis (STEMI), Killip class(≥ III) and plaque property were risk factors for LVSD in ACS patients after PCI (all *P* < 0.05). FIB and albumin were not included in the multivariate analysis because FAR was calculated from them. Multivariate logistic regression showed that FAR, NT-proBNP, NLR, HCY and initial diagnosis (STEMI) were independent predictors of LVSD in ACS patients after adjustment for sex and other potential confounding factors (all *P* < 0.05) .

**Fig. 3:**
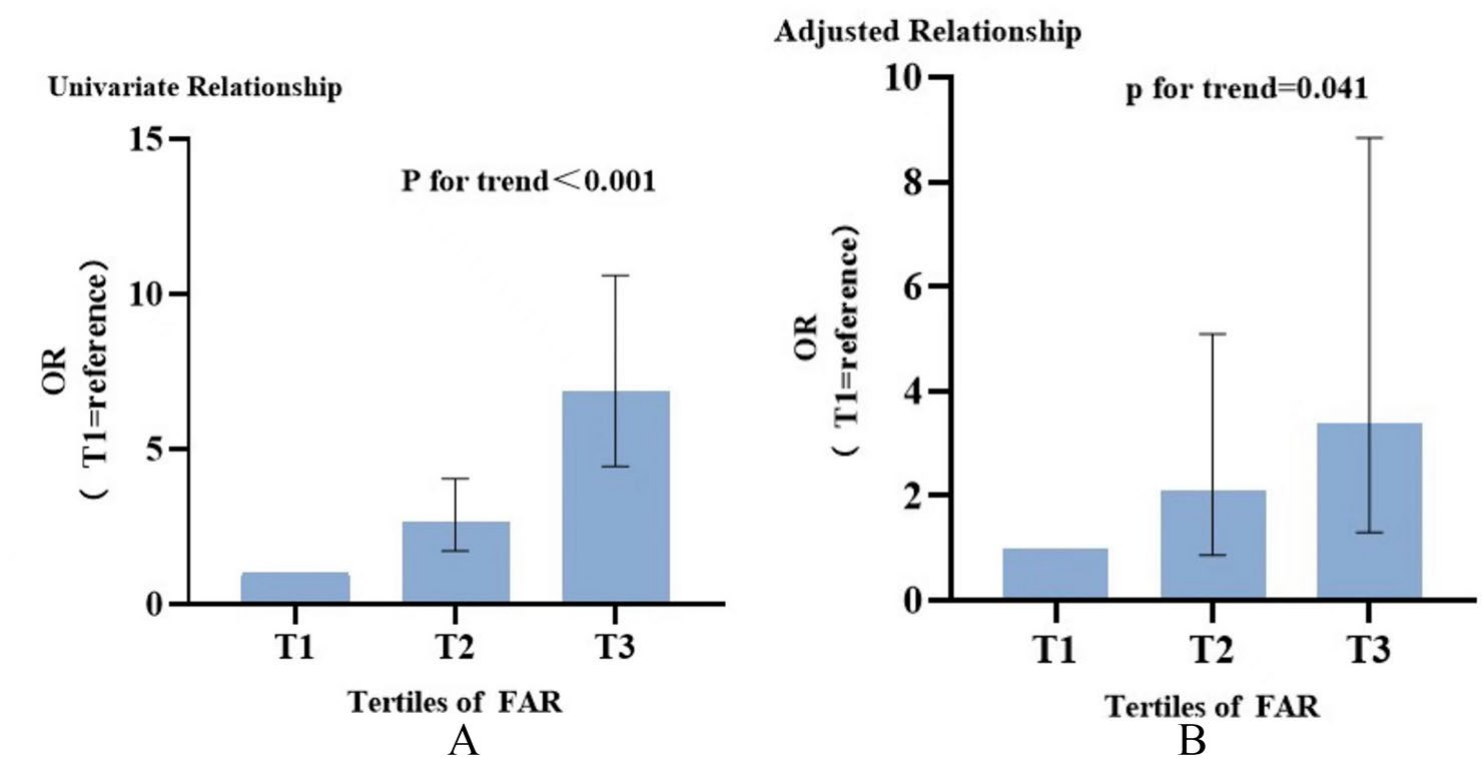
OR of impaired left ventricular ejection fraction according to FAR groups. **A** Crude model. **B** Model4 adjusted. Taking T1 as reference. *OR* Odds ratio, *FAR* fibrinogen-to-albumin ratio

In univariate analysis, FAR as a continuous variable was associated with an OR of 1.037 (95% CI 1.029–1.046; *P* < 0.001). Four models, including variables of statistical significance (*P* < 0.05) and/or clinical importance, were constructed to assess the predictive potential of FAR for LVSD in multivariate logistic regression analysis. Adjustment for multiple confounding variables did not attenuate the correlation and FAR remained to be an independent risk predictor for endpoint (OR 1.030, 95% CI 1.011–1.049; *P* = 0.002) (Table 4). The incidence of the LVSD increased monotonically across the tertiles of FAR in crude model (P for trend ≤ 0.001) (Fig. 3A). Taking T1 as the reference, multivariate analysis revealed that T3 increased the ORs for the incidence of LVSD, while T2 did not reach the statistical significance (T2: OR 2.105, 95% CI 0.869–5.094; T3: OR 3.395, 95% CI 1.303–8.848) (Table 4).

**Table 5:** AUCs of the inflammatory marker values for predicting the occurrence of LVSD

Variables	AUC	95%CI	*P* value	Cut-off	Specificity	Sensitivity
FAR	0.735	0.696–0.774	< 0.001	79.16	0.759	0.596
White blood cells	0.680	0.638–0.723	< 0.001	8.09	0.556	0.728
NLR	0.706	0.664–0.748	< 0.001	3.76	0.632	0.712
MLR	0.688	0.647–0.730	< 0.001	0.21	0.773	0.491
PLR	0.594	0.549–0.638	< 0.001	152.47	0.490	0.674

*AUC* area under receiver operating characteristic curve, *LVSD* left ventricular systolic dysfunction, *CI* confidence interval, *FAR* fibrinogen-to-albumin ratio, *MLR* monocyte-to-lymphocyte ratio, *NLR* neutrophil-to-lymphocyte ratio, *PLR* platelet-to-lymphocyte ratio

### The predictive effect of FAR for LVSD was greater than that of MLR, NLR, and PLR

**Fig. 4:**
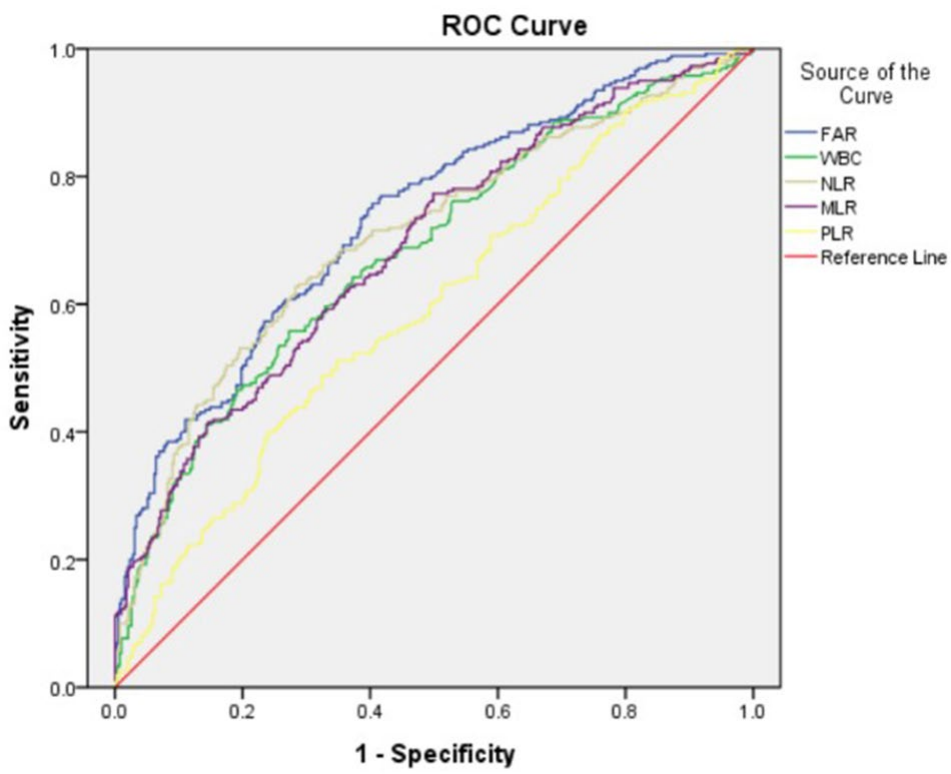
The receiver operating characteristic (ROC) curves based on FAR, white blood cells, NLR, MLR and PLR to predict LVSD in ACS patients after PCI. *FAR* fibrinogen-to-albumin ratio, *WBC* white blood cells, *MLR* monocyte-to-lymphocyte ratio, *NLR* neutrophil-to-lymphocyte ratio, *PLR* platelet-to-lymphocyte ratio

The ROC curves predicting LVSD in ACS patients after PCI are illustrated in Fig. 4. FAR had the highest area under receiver operating characteristic curve (AUC) for prediction of LVSD compared with white blood cells, NLR, MLR and PLR (0.735, 0.680, 0.706, 0.688 and 0.594, respectively) (Table 5). The optimal value of FAR as an indicator for predicting the occurrence of LVSD was 79.16, which yielded a sensitivity of 59.6% and a specificity of 75.9%. The AUCs of FAR, FIB and albumin for the occurrence of LVSD are shown in Additional file 2: Table S2. The AUCs of FAR for predicting the occurrence of LVSD after adjusting for sex and hypertension are shown in Additional file 3: Table S3.

### Subgroup analysis

**Fig. 5:**
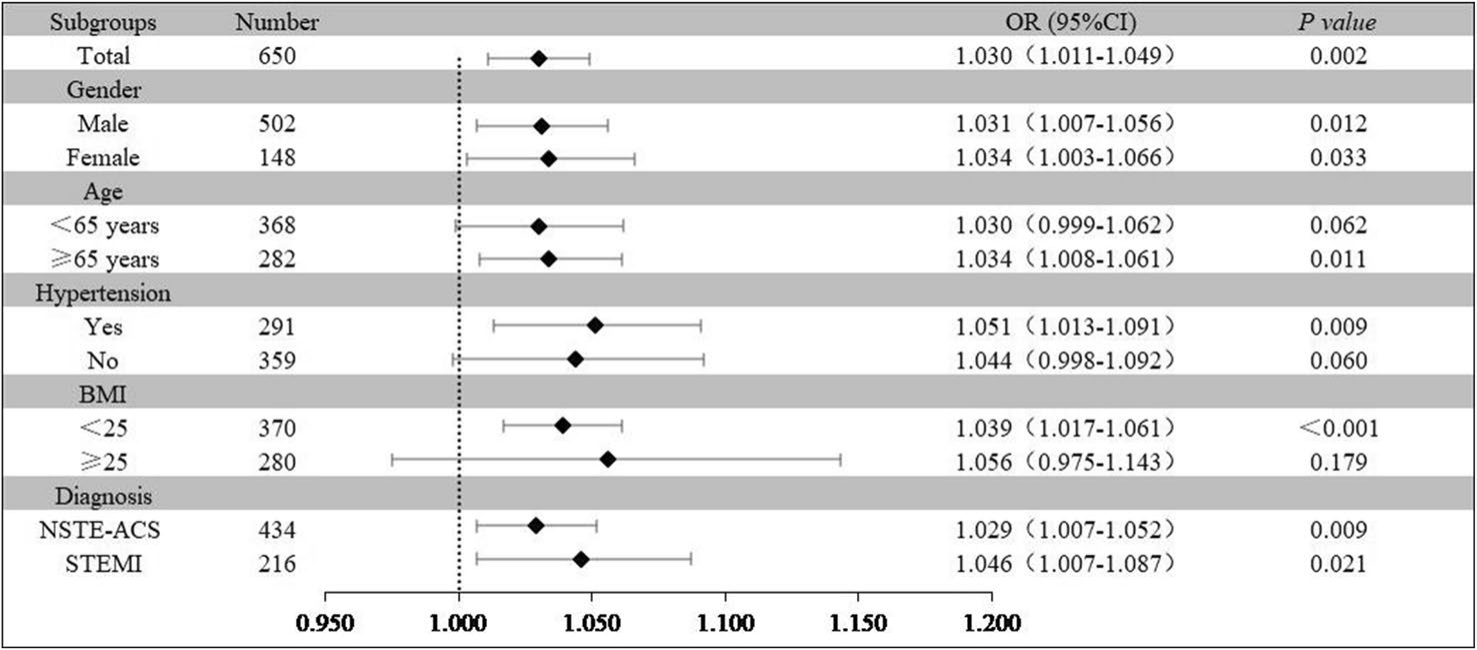
Logistic regression analysis evaluating predictive implication of FAR in various stratifications. OR was evaluated by 1-unit increase of FAR. *OR* Odds ratio, *CI* confidence interval, *BMI* body mass index, NSTE-ACS non-ST-segment elevation acute coronary syndromes, *STEMI* ST-segment elevation myocardial infarction

Relevant clinical variables like sex, age, BMI and clinical diagnosis were subject to post hoc subgroup analyses. The model adjusted in the subgroup analyses comprised all covariates used in Model 4 except for the variables used for stratification. Further evaluation of the predictive value of FAR for LVSD was performed in different subclasses. Increased FAR (per 1 unit) was consistently related to LVSD in various subgroups, including female or male, age ≥ 65 years, BMI < 25 kg/m^2^, with hypertension, NSTE-ACS or STEMI (Fig. 5). However, the results were not similar in patients aged below 65 years and patients without hypertension.

### The nomogram model

**Fig. 6:**
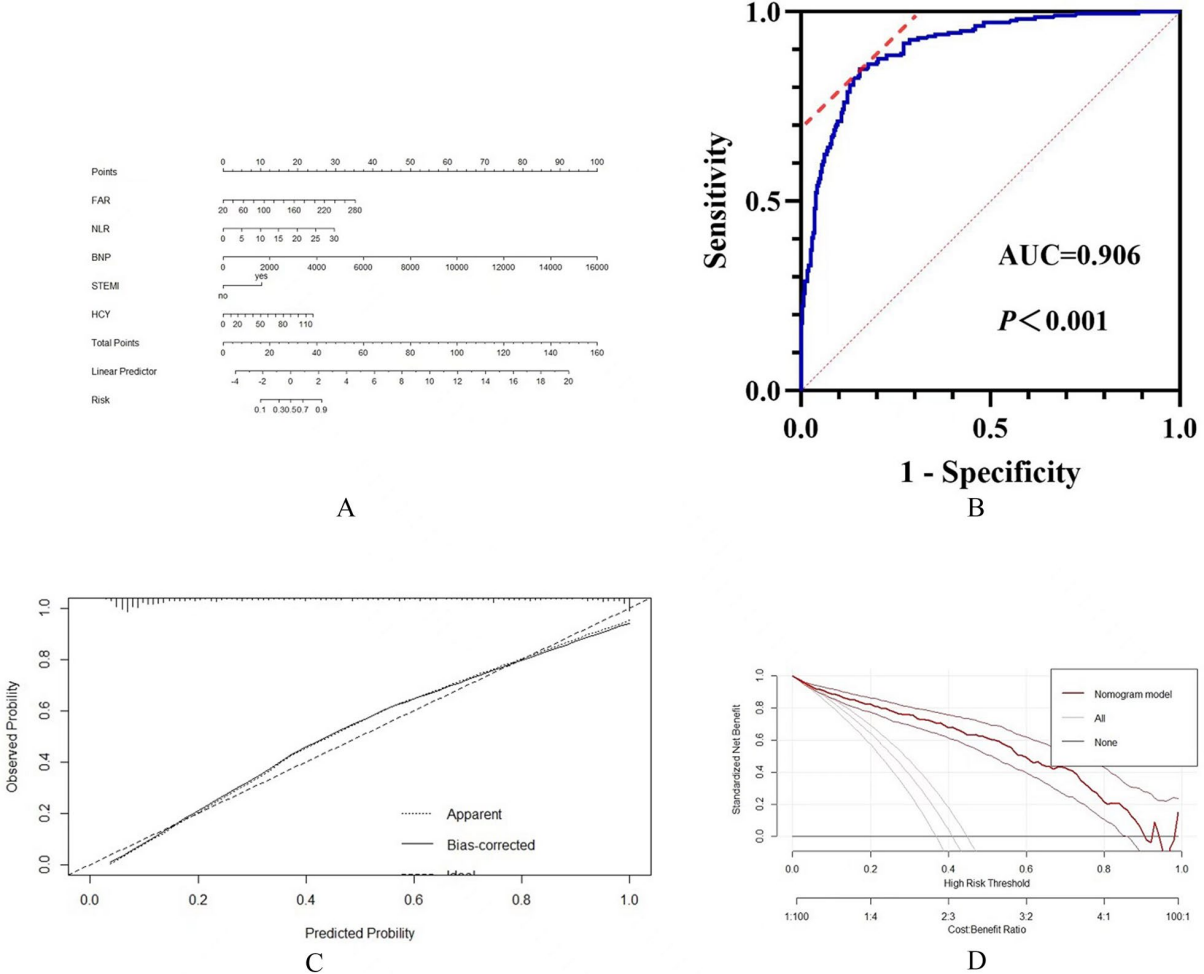
Construction and evaluation of nomogram. **A** Nomogram for prediction of LVSD. **B** C-index curves of the model. **C** Calibration curves of the model. **D** DCA curves of the model

A nomogram was constructed to predict LVSD based on the final regression analysis (Fig. 6A). Furthermore, the AUC of the nomogram for LVSD were 0.906 (95%CI 0.881–0.932) in patients with ACS, indicting strong discrimination (Fig. 6B). A calibration curve of the nomogram is presented in Fig. 6C. The DCA indicated that the model showed better clinical benefit (Fig. 6D).

## Discussion

In the present study, the relationship between FAR and left ventricular systolic function was investigated in patients with ACS who underwent PCI with stent implantation. Patients with left ventricular systolic dysfunction had significantly higher FAR values than patients with preserved LVEF in the study population. FAR was a strong indicator of left ventricular dysfunction even after adjustment for confounders. In addition, the ROC curve demonstrated the predictive power of FAR was greater than that of NLR, followed by MLR, white blood cells and PLR for LVSD in ACS patients. To our knowledge, this is the first study exploring the predictive role of FAR to the LV function between ACS patients after PCI. These findings supported that inflammation indicators were effective markers for predicting LVSD in ACS patients. In addition, the results of this study may contribute to better risk stratification and management of patients with ACS.

Fibrinogen (FIB), an acute-phase protein, is synthesized primarily in hepatocytes and plays a crucial role in the physiology and pathophysiology of coagulation and inflammation [14]. Fibrinogen biosynthesis increases rapidly during the acute phase of inflammation, such as bacterial infection, severe trauma and surgery [15]. Elevated plasma fibrinogen levels are also involved in chronic, low-grade inflammatory processes, activation of platelets, adhesion molecule expression upregulation, stimulation of angiogenesis and macrophages infiltration enhancement, which consequently aggravate atherosclerotic plaque progression [16]. Increased plasma fibrinogen concentration been confirmed the cause of the development of atherosclerotic lesions. Numerous observational studies identified that increased plasma fibrinogen concentrations were closely associated with CVD. Yuan et al. reported that plasma FIB was independently associated with long-term risk of all-cause and cardiac mortality in CAD patients after PCI [17]. Jiang et al. indicated fibrinogen concentration was associated with 2-year all-cause mortality in patients undergoing PCI [18]. Many cardiovascular risk factors can reversely lead to increased plasma concentration of fibrinogen, like age, diabetes, hypertension, obesity, lipid disorders, metabolic syndrome, smoking and alcohol consumption [19]. Albumin is synthesized in the liver, and the synthesis ability is affected by both nutrition and inflammation condition [20]. Malnutrition and inflammation are considered to play a major role in occurrence of hypoalbuminemia. Serum albumin has many physiological properties, such as anti-inflammatory activity, antioxidant, anticoagulant, antiplatelet aggregation and maintenance of capillary membrane stability [21]. Evidence has emerged that hypoalbuminemia is a powerful prognostic marker in the general population and in patients with cardiovascular diseases. After adjustment for traditional cardiovascular risk factors, serum albumin levels remained inversely associated with ischemic heart disease, heart failure and stroke [22–24]. Also, hypoalbuminemia is a powerful predictor of the cardiovascular prognosis in patients with CVD. A previous study had shown that lower serum albumin levels were associated with adverse cardiac events in patients with CAD after PCI [25]. Given that both plasma FIB and albumin showed strong correlation with adverse cardiovascular events, subsequent studies are warranted to evaluate whether FAR could be helpful in identifying high-risk populations in ACS patients undergoing PCI.

Since fibrinogen and albumin are positively and negatively correlated with systemic inflammation, respectively, researchers have proposed the hypothesis that FAR may be more closely related to inflammation than fibrinogen or albumin alone. Previous studies have confirmed the combination of fibrinogen and albumin parameters represent a more reliable and efficient indicator for the prognosis of multiple tumors and cardiovascular events than individual parameter separately (A–E). FAR was shown to be an independent predictor of the presence and severity of CAD among angina patients [26]. Oğuz et al. demonstrated that FAR was significantly associated with SYNTAX score in STEMI patients after PCI [27]. Furthermore, Xiao et al. analyzed 475 patients with STEMI and determined that the FAR was an independent prognostic factor for all-cause mortality in the population [28]. Recent research also reported that the FAR was an independent predictor of long-term outcomes in patients with NSTE-ACS who underwent PCI [29]. Consistent with the results of the above studies, FAR has been shown to be more powerful than fibrinogen or albumin alone in predicting the prognosis of patients with malignant tumors. Qiang et al. indicated that FAR was a novel prognostic indicator for patients with stage IB-IIA cervical cancer [30]. High FAR had been shown to be inversely associated with overall survival for locally advanced or metastatic pancreatic cancer [31]. In addition, FAR was reported to be a valuable marker for predicting long-term adverse prognosis in patients with gastric cancer treated with first-line chemotherapy, and its prognostic value was superior to that of fibrinogen or albumin alone [32].

Recent studies revealed that elevated NLR was an independent predictor for LVSD in ACS patients [33, 34]. NLR was demonstrated negatively associated with LVEF in patients with NSTE-ACS [33]. Orhan et.al found that NLR was a sensitive and specific predictor of impaired LV systolic dysfunction [34]. Adem et al. reported high PLR was a strong and independent predictor for LVSD in NSTE-ACS patients [35]. It is previously shown that elevated WBC levels are an independent predictor for the occurrence of LVSD after ACS regardless of several confounding factors [36]. Consistent with these results, we also found that NLR was an independent predictor of LVSD after adjusting for multiple covariates in ACS patients undergoing PCI.

Left ventricular dysfunction has been proved as the arguably powerful predictor of morbidity and mortality in ACS patients [4]. There are multiple mechanisms contributing to adverse left ventricular remodeling after acute myocardial infarction, such as large infarct size, excessive inflammatory response, irreversible microvascular disturbance, extracellular matrix changes, collagen deposition, fibroblast aggregation, eccentric hypertrophy, oxidative stress and neurohormonal activation [37, 38]. Our findings implied that elevated FAR may be partly involved in potential mechanism of left ventricular remodeling after ACS, resulting in decreased LVEF. Previous studies showed that higher FAR levels were significantly and independently related to the presence of angiographic coronary slow flow and no-reflow [39, 40]. The occurrence of coronary no-reflow may be associated with diffuse atherosclerosis, increased systemic inflammatory load, platelet dysfunction and impaired endothelial function, leading to coronary microvascular dysfunction. Therefore, we proposed that higher FAR may have caused worse microvascular perfusion, thereby affecting left ventricular functions. Considering these findings, it is reasonable to further investigate the underlying mechanisms for FAR in left ventricular remodeling.

The present study has some limitations. Firstly, the retrospective study was based on a single-center trial with a limited sample size and may not be generalized to other cohorts. Secondly, residual confounding by other unmeasured covariates cannot be excluded despite the attempt to perform potential risk factors adjustment. Finally, the measurement of echocardiogram was performed only once within 24 h after admission and may have failed to measure changes in LVEF after revascularization. Further multi-centric studies with larger populations are needed to clarify potential association between FAR in patients with left ventricular systolic dysfunctions.

## Conclusions

FAR is an affordable and reliable predictor of LV systolic dysfunction in ACS patients undergoing PCI and the predictive power of FAR is greater than that of MLR, NLR, and PLR. Thus, the practice of using FAR on admission may help identify high-risk patients and relevant treatments.

## Supplementary Information

**Additional file 1: Table S1**. Univariate and multivariate analysis and predictors of LVSD in ACS patients.

**Additional file 2: Table S2**. AUCs of FAR, FIB and albumin predicting the occurrence of LVSD.

**Additional file 3: Table S3.** AUCs of FAR predicting the occurrence of LVSD after adjusting for sex and hypertension.

## Data Availability

All data used and analyzed in this study are available from the corresponding author on reasonable request.

## Abbreviations

CAD: Coronary artery disease
ACS: Acute coronary syndrome
LVSD: Left ventricular systolic dysfunction
LVEF: Left ventricular ejection fraction
NLR: Neutrophil-to-lymphocyte ratio
PLR: Platelet-to-lymphocyte ratio
MLR: Monocyte-to-lymphocyte ratio
PCI: Percutaneous coronary intervention
UA: Unstable angina
NSTEMI: Non-ST-segment elevation myocardial infarction
STEMI: ST-segment elevation myocardial infarction
FIB: Fibrinogen
CVD: Cardiovascular disease
