# Supplementary material for: Predictive impact of fibrinogen-to-albumin ratio (FAR) for left ventricular dysfunction in acute coronary syndrome: a cross-sectional study
Source: Eur J Med Res. 2023 Feb 8;28:68. doi: 10.1186/s40001-023-01029-2 (PMC9906889; doi:10.1186/s40001-023-01029-2)
Supplement: Supplementary file 1 — Additional file 1: Table S1. Univariate and multivariate analysis and predictors of LVSD in ACS patients. [file 40001_2023_1029_MOESM1_ESM.docx]

Additional file 1: Table S1 Univariate and multivariate analysis and predictors of LVSD in ACS patients

|  | Univariate analysis | | |  | Multivariate analysis | | |
| --- | --- | --- | --- | --- | --- | --- | --- |
|  | OR | 95% CI | *P* value |  | OR | 95% CI | *P* value |
| FAR | 1.037 | 1.029-1.046 | <0.001 |  | 1.038 | 1.020-1.057 | <0.001 |
| Sex | 1.800 | 1.213-2.669 | 0.003 |  |  |  |  |
| Heart rate | 1.028 | 1.015-1.041 | <0.001 |  |  |  |  |
| SBP | 0.979 | 0.971-0.987 | <0.001 |  |  |  |  |
| Smoking | 1.947 | 1.412-2.684 | <0.001 |  |  |  |  |
| Hypertension | 0.548 | 0.399-0.753 | <0.001 |  |  |  |  |
| NT-proBNP | 1.001 | 1.001-1.002 | <0.001 |  | 1.001 | 1.001-1.001 | <0.001 |
| White blood cells | 1.264 | 1.189-1.343 | <0.001 |  |  |  |  |
| NLR | 1.288 | 1.209-1.372 | <0.001 |  | 1.201 | 1.083-1.332 | 0.001 |
| MLR | 158.386 | 43.275-579.698 | <0.001 |  |  |  |  |
| PLR | 1.005 | 1.002-1.007 | <0.001 |  |  |  |  |
| hs-CRP | 1.126 | 1.071-1.184 | <0.001 |  |  |  |  |
| ALT | 1.014 | 1.007-1.021 | <0.001 |  |  |  |  |
| AST | 1.011 | 1.008-1.014 | <0.001 |  |  |  |  |
| Albumin | 0.866 | 0.833-0.900 | <0.001 |  |  |  |  |
| Creatinine | 1.011 | 1.003-1.020 | 0.008 |  |  |  |  |
| Cystatin C | 2.100 | 1.287-3.428 | 0.003 |  |  |  |  |
| eGFR | 0.985 | 0.974-0.996 | 0.007 |  |  |  |  |
| Na^+^ | 0.886 | 0.843-0.931 | <0.001 |  |  |  |  |
| Ca^2+^ | 0.016 | 0.005-0.055 | <0.001 |  |  |  |  |
| Homocysteine | 1.038 | 1.024-1.052 | <0.001 |  | 1.052 | 1.026-1.077 | <0.001 |
| PT | 1.621 | 1.307-2.010 | <0.001 |  |  |  |  |
| PTA | 0.971 | 0.959-0.983 | <0.001 |  |  |  |  |
| INR | 135.373 | 16.096-1138.560 | <0.001 |  |  |  |  |
| APTT | 1.072 | 1.039-1.107 | <0.001 |  |  |  |  |
| FIB | 2.497 | 2.005-3.110 | <0.001 |  |  |  |  |
| D-dimer | 1.649 | 1.248-2.179 | <0.001 |  |  |  |  |
| FDP | 1.201 | 1.064-1.356 | 0.003 |  |  |  |  |
| Triglycerides | 0.663 | 0.529-0.830 | <0.001 |  |  |  |  |
| Lp(a) | 1.001 | 1.001-1.002 | <0.001 |  |  |  |  |
| Gensini score | 1.012 | 1.008-1.016 | <0.001 |  |  |  |  |
| Initial diagnosis |  |  |  |  |  |  |  |
| NSTE-ACS | 1(reference) |  |  |  |  |  |  |
| STEMI | 9.777 | 6.693-14.282 | <0.001 |  | 5.660 | 2.825-11.340 | <0.001 |
| Killip class |  |  |  |  |  |  |  |
| <Ⅲ | 1(reference) |  |  |  |  |  |  |
| ≥Ⅲ | 2.796 | 1.627-4.805 | <0.001 |  |  |  |  |
| Target vessel territory |  |  |  |  |  |  |  |
| LAD | 1.630 | 1.165-2.279 | 0.004 |  |  |  |  |
| Number of stents |  |  |  |  |  |  |  |
| ≥3 | 1.412 | 1.004-1.987 | 0.047 |  |  |  |  |
| Plaque property, n (%) |  |  |  |  |  |  |  |
| Calcification lesions | 0.627 | 0.423-0.931 | 0.021 |  |  |  |  |
| Diffuse lesions | 0.651 | 0.451-0.940 | 0.022 |  |  |  |  |
| Thrombus | 2.327 | 1.101-4.917 | 0.027 |  |  |  |  |
| Chronic total occlusions | 2.006 | 1.289-3.124 | 0.002 |  |  |  |  |

*SBP* systolic blood pressure, *hs-CRP* high-sensitivity C-reactive protein, *NT-proBNP* N-terminal pro-B type natriuretic peptide, *FAR* fibrinogen-to-albumin ratio, *MLR* monocyte-to-lymphocyte ratio, *NLR* neutrophil-to-lymphocyte ratio, *PLR* platelet-to-lymphocyte ratio, *ALT* Alanine transaminase, *AST* Aspartate aminotransferase, eGFR estimated glomerular filtration rate, *Na^+^* serum sodium, *Ca^2+^* serum calcium, *PT* prothrombin time, *PTA* prothrombin time activity, *INR* international normalized ratio, *APTT* activated partial thromboplastin time, *FIB* fibrinogen, *FDP* fibrinogen degradation products, *Lp(a)* Lipoprotein(a), *NSTE-ACS* non-ST-segment elevation acute coronary syndrome, *STEMI* ST-segment elevation myocardial infarction, *LAD* left anterior descending artery.
