# Supplementary material for: Predictive impact of fibrinogen-to-albumin ratio (FAR) for left ventricular dysfunction in acute coronary syndrome: a cross-sectional study
Source: Eur J Med Res. 2023 Feb 8;28:68. doi: 10.1186/s40001-023-01029-2 (PMC9906889; doi:10.1186/s40001-023-01029-2)
Supplement: Supplementary file 2 — Additional file 2: Table S2. AUCs of FAR, FIB and albumin predicting the occurrence of LVSD. [file 40001_2023_1029_MOESM2_ESM.docx]

Additional file 2: Table S2 AUCs of FAR, FIB and albumin predicting the occurrence of LVSD

| Variables | AUC | 95%CI | *P* value | Cut-off | Specificity | Sensitivity |
| --- | --- | --- | --- | --- | --- | --- |
| FAR | 0.735 | 0.696-0.774 | <0.001 | 79.16 | 0.759 | 0.596 |
| FIB | 0.700 | 0.659-0.741 | <0.001 | 3.34 | 0.659 | 0.645 |
| Albumin | 0.666 | 0.622-0.710 | <0.001 | 37.1 | 0.387 | 0.887 |

*AUC* area under receiver operating characteristic curve, *LVSD* left ventricular systolic dysfunction, *CI* confidence interval, *FAR* fibrinogen-to-albumin ratio, *FIB* fibrinogen.
