# Supplementary material for: Predictive impact of fibrinogen-to-albumin ratio (FAR) for left ventricular dysfunction in acute coronary syndrome: a cross-sectional study
Source: Eur J Med Res. 2023 Feb 8;28:68. doi: 10.1186/s40001-023-01029-2 (PMC9906889; doi:10.1186/s40001-023-01029-2)
Supplement: Supplementary file 3 — Additional file 3: Table S3. AUCs of FAR predicting the occurrence of LVSD after adjusting for sex and hypertension. [file 40001_2023_1029_MOESM3_ESM.docx]

Additional file 3: Table S3 AUCs of FAR predicting the occurrence of LVSD after adjusting for sex and hypertension

| Variables | FAR |  |  |  |
| --- | --- | --- | --- | --- |
|  | Male | Female | HT | Non-HT |
| AUC | 0.739 | 0.772 | 0.741 | 0.737 |
| 95%CI | 0.696-0.783 | 0.685-0.858 | 0.686-0.797 | 0.682-0.792 |
| *P* value | <0.001 | <0.001 | <0.001 | <0.001 |

*AUC* area under receiver operating characteristic curve, *LVSD* left ventricular systolic dysfunction, *CI* confidence interval, *FAR* fibrinogen-to-albumin ratio, *HT* hypertension.
